# Hyperthermia Treatment Planning Including Convective Flow in Cerebrospinal Fluid for Brain Tumour Hyperthermia Treatment Using a Novel Dedicated Paediatric Brain Applicator

**DOI:** 10.3390/cancers11081183

**Published:** 2019-08-15

**Authors:** Gerben Schooneveldt, Hana Dobšícek Trefná, Mikael Persson, Theo M. de Reijke, Klas Blomgren, H. Petra Kok, Hans Crezee

**Affiliations:** 1 Department of Radiotherapy, Amsterdam UMC, University of Amsterdam, 1105 AZ Amsterdam, The Netherlands; 2 Department of Electrical Engineering, Chalmers University of Technology, 41296 Gothenburg, Sweden; 3 Department of Urology, Amsterdam UMC, University of Amsterdam, 1105 AZ Amsterdam, The Netherlands; 4 Department of Women’s and Children’s Health, Karolinska Institutet, 17164 Stockholm, Sweden; 5 Department of Pediatric Oncology, Karolinska University Hospital, 17164 Stockholm, Sweden

**Keywords:** hyperthermia treatment, treatment planning, computational fluid dynamics, cerebrospinal fluid, brain malignancies, medulloblastoma

## Abstract

Hyperthermia therapy (40–44 °C) is a promising option to increase efficacy of radiotherapy/chemotherapy for brain tumours, in particular paediatric brain tumours. The Chalmers Hyperthermia Helmet is developed for this purpose. Hyperthermia treatment planning is required for treatment optimisation, but current planning systems do not involve a physically correct model of cerebrospinal fluid (CSF). This study investigates the necessity of fluid modelling for treatment planning. We made treatments plans using the Helmet for both pre-operative and post-operative cases, comparing temperature distributions predicted with three CSF models: a convective “fluid” model, a non-convective “solid” CSF model, and CSF models with increased effective thermal conductivity (“high-*k*”). Treatment plans were evaluated by *T*_90_, *T*_50_ and *T*_10_ target temperatures and treatment-limiting hot spots. Adequate heating is possible with the helmet. In the pre-operative case, treatment plan quality was comparable for all three models. In the post-operative case, the high-*k* models were more accurate than the solid model. Predictions to within ±1 °C were obtained by a 10–20-fold increased effective thermal conductivity. Accurate modelling of the temperature in CSF requires fluid dynamics, but modelling CSF as a solid with enhanced effective thermal conductivity might be a practical alternative for a convective fluid model for many applications.

## 1. Introduction

Hyperthermia treatment, i.e., heating tumours to fever range temperatures (ca. 40–44 °C) for a prolonged time (typically 1 h), has been proven to increase response rate and survival for a wide range of tumours in patients treated with radiotherapy [1,2,3,4] or chemotherapy [5,6,7]. For example, a randomized controlled trial comparing radiotherapy with and without hyperthermia in the treatment of cervical cancer, showed a significantly improved 12-year local control (56% vs. 37%) and 12-year overall survival (37% vs. 20%) in the hyperthermia arm [8]. Similarly, a randomized controlled trial comparing chemotherapy with and without hyperthermia in the treatment of non-muscle invasive bladder cancer, demonstrated that adding hyperthermia significantly improved 10-year disease free survival from 15% to 53% [9].

Brain tumours might particularly benefit from this synergistic treatment as radiotherapy leads to severe late toxicities, especially in children [10]. The fact that brain tumours are relatively common in children makes tackling this problem even more urgent. Moreover, many chemotherapeutic agents do not adequately permeate through the blood brain barrier (BBB). Hyperthermia may mitigate both problems. As one of the most potent radiosensitizers known, it allows for lower radiation doses with equal treatment outcome, and no increased long term toxicities [11,12]. It has also been known to temporarily open the BBB [13,14,15]. Kiyatkin et al. showed that the BBB in rats appears to become permeable at 38.5 °C, reaching full permeability at 41 °C [16], suggesting that relatively mild hyperthermia might suffice to enhance treatment efficacy. Another study reported that the BBB in rats remains impermeable below 40.3 °C, but is fully permeable at 42.5 °C [17].

There are various types of malignant (primary) brain tumours, such as glioblastoma multiforme (most common in adults) and medulloblastoma (most common in children). Clinical application of hyperthermia for glioblastoma [18,19,20,21] has shown promising results; e.g., Sneed et al. [18] reported a two-year survival rate of 31% in the arm with hyperthermia versus 15% in the arm without hyperthermia. Byun et al. [22] reported, in a single-arm study in metastatic brain tumours, a 3-year local recurrence rate of 15,8% after intra-operative hyperthermia, compared to 34% for a control group. However, large-scale randomized controlled trials are lacking. Hyperthermia as an adjuvant treatment for medulloblastoma has not yet been reported, but may similarly be expected to have a positive effect. Nevertheless, heating the brain is not without difficulties. In general, the challenge is to get a sufficiently high temperature in the tumour for a prolonged time, without excessive temperature increase in other parts of the brain. As the brain is very sensitive to high temperatures [23,24], hyperthermia treatment of the brain requires stricter temperature control than treatments in e.g., the pelvic area. In addition, patients need to be awake and able to report neurological complaints related to excessive temperatures in normal brain tissue. 

There are several methods to raise the temperature in the brain, each with its own advantages and drawbacks [25]. High-intensity focussed ultrasound (HIFU) can be an accurate treatment option that may be combined with MR imaging (in particular MR thermometry), but the cranial bone often makes HIFU delivery difficult or even impossible. Nevertheless, promising results have been shown in mice and rats [26,27]. Non-thermal focussed ultrasound has been used in combination with intravenously injected microbubbles to temporarily disrupt the BBB in a clinical feasibility study [28]. Magnetic nanoparticles are relatively easy to heat, but the temperature is difficult to control, and the particles may cause severe artefacts in MR imaging, both for MR-based temperature control and follow-up imaging [21]. Interstitial hyperthermia may obtain acceptable temperatures in the bulk of the macroscopic tumour without creating unacceptable hot spots in healthy tissue, but may be inadequate for heating the tumour infiltrations in surrounding normal brain tissue [19]. In practice, the implantation of the required catheters is only justifiable when the patient also receives brachytherapy, using the same catheters. Loco-regional microwave hyperthermia, on the other hand, should be able to heat a larger portion of the brain, optionally including a margin around the tumour; such a margin may be important because of diffuse infiltration (e.g., in the case of gliomas) or incomplete resection (e.g., in the case of medulloblastomas, where about 50% of resections is incomplete [29]), and may additionally result in better tumour coverage. Adequate power steering is important to avoid the risk of causing hot spots in healthy tissue, potentially limiting overall treatment quality. 

Although clinical application of hyperthermia for brain tumours has been limited to glioblastoma multiforme, the most prevalent brain tumour in adults, many of the considerations (opening of the BBB, radio-sensitisation, chemo-sensitisation) are equally valid for the treatment of medulloblastomas, the most common brain tumour in children. Even low doses of ionizing radiation to the brain can cause intellectual impairment [30,31,32] as well as perturbed growth [30,33] and puberty [34] and, consequently, a radiosensitising treatment that may lead to lower radiation doses, is desired [10]. Adding hyperthermia may lead to the same effective dose with a lower physical dose, resulting in fewer side effects [12,35].

**Figure 1:**
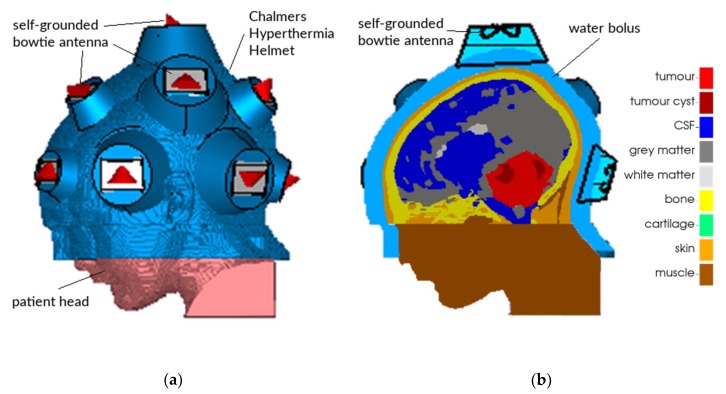
Model of the Chalmers Hyperthermia Helmet. Up to 16 self-grounded bowtie antennas with a frequency range of 300–900 MHz are mounted around the skull. Phase and amplitude can be optimised individually for each antenna, creating a power focus to be steered to the tumour. (**a**) Outside view; (**b**) cross-section of a patient model, showing a large medulloblastoma in red and the water bolus in light blue.

Despite these promising possibilities of adjuvant hyperthermia treatment for paediatric medulloblastoma, current standard of care is limited to post-operative radiotherapy in combination with chemotherapy, although also chemotherapy-related toxicity remains a major concern [36]. The absence of trials investigating the benefit of adding hyperthermia in the treatment of paediatric medulloblastoma, may be partly due to the technical challenges in administering adequate hyperthermia treatment in these patients. In order to enable paediatric brain hyperthermia treatment, a microwave applicator, tentatively dubbed the Chalmers Hyperthermia Helmet, was developed tailored for children, comprising up to 16 antennae operating at 430–900 MHz [37,38,39,40]. A schematic depiction of the second generation of the Chalmers Hyperthermia Helmet is shown in Figure 1. The applicator comprises two arrays of self-grounded bow-tie antennas [39] of two different dimensions and operated at 300–550 MHz and 430–900 MHz, respectively. Between the patient’s skin and the antennas, one or more water boli are provided, to couple the electromagnetic field generated by the antennas into the patient and to cool the patient’s skin. However, achieving a clinically acceptable temperature distribution may be challenging. This is partly caused by the very high perfusion of the white and grey matter, quickly removing heat and hence requiring relatively strong EM-fields; and partly by the presence of the cerebrospinal fluid (CSF). Due to its high electric conductivity (σ > 2.0 S/m), the CSF absorbs EM radiation very efficiently and is thus easily heated, quickly leading to treatment-limiting hot spots. Since the CSF is a fluid, this heat can be transported over relatively large distances with the fluid flow. Therefore, CSF has a significant influence on the temperature distribution in a large part of the brain. 

However, current hyperthermia treatment planning systems do not model fluid dynamics in a physically correct manner, and do not take the effects of fluid flow into account, except, to some extent, blood perfusion. Blood flow through small vessels (i.e., blood perfusion of various tissues) is typically included with a greatly simplified model like Pennes’ bioheat equation [41], and some systems also model individual large blood vessels using so-called discrete vasculature models [42,43]; however, the effects of flow in larger contiguous fluid volumes are unaccounted for. Heat transport in such fluids has two components: conductive (which is similar to solid tissues) and convective (which is associated with the bulk motion of the fluid). In body fluids with sufficient volume, such as blood in large blood vessels, urine in the bladder, or CSF in the brain, convective heat transport is usually dominant.

For CSF specifically, heat is gained through direct absorption from the EM field and through heat exchange with directly neighbouring tissues warmer than the CSF; and heat is lost through heat exchange with tissues with a lower temperature, which in turn remove excess heat through heat exchange with blood vessels. This results in temperature differences in the CSF, which give rise to convective fluid flows, with a net effect of transporting heat upwards (against the direction of gravity). Treatment planning systems that do not model fluid flow may, as a consequence, tend to overestimate CSF-induced hot spots, and underestimate the heat distribution by the CSF.

Recently, we developed a thermodynamic fluid model as an extension to our existing treatment planning system, Plan2Heat [44]. The direct motivation was to model the influence of urine, which is also a relatively large contiguous fluid volume, on the temperature distribution in and around the urinary bladder during pelvic hyperthermia treatments. This fluid dynamics modelling module has been validated experimentally, showing an accuracy of ~0.1 °C [45,46,47], and has been applied successfully for (retrospective) hyperthermia treatment evaluation of bladder cancer patients [48,49].

The aim of our current research is to assess the effect of CSF flow on the temperature distribution in the human brain during microwave hyperthermia treatment using the Chalmers Hyperthermia Helmet by numerical simulations including fluid dynamics modelling. Results are compared to those obtained with standard solid models and will show whether fluid modelling is important for accurate treatment planning.

## 2. Materials and Methods

### 2.1. Patient Model and Electromagnetic Field Simulations

An MRI scan with a 1 × 1 × 1 mm resolution was obtained from an actual patient, a 13-year old boy with a large medulloblastoma (92 mL); in the context of the current proof-of-concept study, this may be considered a valid model for various brain tumours. The scan was manually segmented by a clinician into thirteen tissue types; the most relevant ones of which are tumour, tumour cyst, CSF, white matter, grey matter, muscle, and bone. As can be seen in Figure 1b and Figure 10, only part of the head was segmented. The section outside the treatment volume, i.e., the part of the head below the tumour that is not covered by the applicator, was modelled as muscle. The tissue parameters used in the simulations are based on literature values and are listed in Table 1 [50,51,52,53]. Normothermal perfusion coefficients were used, except for muscle, for which a four-fold increased hyperthermic perfusion rate of 3.6 kg/(m^3^ s) was used reflecting the enhanced muscle perfusion levels in response to hyperthermic temperature elevations [53]. As in practice, hyperthermia treatments for medulloblastoma patients would most likely be delivered post-operatively, we also created a ‘post-operative mesh’, in which the tumour has been excised and the region was filled with CSF. This post-operative mesh was obtained by labelling and treating all tumour and tumour cyst voxels as CSF. Although realistic deformations might be less simplistic, this procedure provides a good test case for the purpose of this paper. All simulations were performed for both the original, or ‘pre-operative’ mesh, and for the post-operative mesh.

A model of the Chalmers Hyperthermia Helmet comprising a water bolus at 10 °C and 14 antennas operating at 450 MHz was created around the segmented head model [40,54]. The applicator comprises two arrays of self-grounded bow-tie antennas [39] of two different dimensions and operated at 300–550 MHz and 430–900 MHz, respectively. The frequency considered in this study is thus a common frequency for all fourteen antennas. For each antenna, the radiated electromagnetic field was computed, with unit amplitude and zero phase, using CST Studio Suite (Dassault Systèmes, Vélizy-Villacoublay, France). Subsequently, the amplitude and phase for each of the antennas were optimised to create a power focus in the tumour region, using a time-reversal focusing method [55].

### 2.2. Temperature Simulations

The computation of the temperature distribution uses the same segmentation and is computed based on this electromagnetic field distribution using a finite volume thermodynamic fluid model. This model was built in OpenFOAM [56] and implements Pennes’ bioheat equation [41] in the solid tissue regions, and uses the Boussinesq approximation to the Navier–Stokes equations for the fluid (CSF) regions [57], which reads:

(1) $$\rho \frac{\partial \vec{v}}{\partial t}+\rho \;\nabla \cdot \left(\vec{v}\vec{v}\right)=\mu \;\nabla^{2}\vec{v}-\nabla p+\rho_{k}\;\vec{g}.\;\;(\mathrm{kg}/\left(m^{2}{\;s}^{2}\right))$$

The left-hand side expresses the co-moving change over time *t* (s) to the velocity $\vec{v}$ (m/s) of a fluid element with (constant) mass density ρ (kg/m^3^). The right-hand side describes the forces acting on the fluid element: the first term is the ‘friction’ between neighbouring fluid elements quantified by the dynamic viscosity μ (Pa s), the second term is the spatial difference in pressure *p* (Pa). The third term drives the convection and includes the gravitational acceleration $\vec{g}$ (m/s) and the (non-constant) kinematic density ρ_k_ (kg/m), which is assumed to depend linearly on the temperature. The equation governing temperature change in the fluid compartment is:

(2) $$C_{p}\;\rho \;\frac{\partial T}{\partial t}=k\;\nabla^{2}T-C_{p}\;\rho \;\left(\vec{v}\cdot \nabla T\right)+\frac{1}{2}\sigma \;E^{2},\;\;\left(W/m^{3}\right)$$

with *C_p_* (J/(kg K)) the specific heat per mass, *k* (W/(m K)) the thermal conductivity, σ (S/m) the electric conductivity, *E* (V/m) the electric field strength, and all other variables as in Equation (1). The reader is referred to earlier publications for full details [46,48]. 

Meshes for thermal simulations in OpenFOAM were created and the resolution in the solid regions was kept at 1 × 1 × 1 mm; in the fluid regions, these (1 mm)^3^ cubes were subdivided into six identical pyramids by adding a vertex at the centre of the cube, in order to increase the computational stability. For both the pre-operative case and the post-operative case, we evaluated three different models to simulate the thermal behaviour of CSF:

1. fluid: the CSF was modelled using fluid dynamics, including convective flow;
2. solid: the CSF was modelled as a solid, similar to current practice in available hyperthermia treatment planning systems; and
3. high-*k*: the CSF was modelled as a solid with an *n*-fold increased thermal conductivity *k*_eff_, with *k*_eff_/*k* = 2, 5, 10, 20, 50 and 100.

The comparison of these cases allows to evaluate the influence of fluid dynamics on the predicted temperature distributions. Fluid dynamics modelling is relatively expensive in terms of computational costs. The third case with enhanced effective thermal conductivity *k*_eff_ approximates the effect of heat transport by fluid flow in a relatively simple and computationally efficient model, similar to that described by Yuan et al. [58], allowing easy integration in a clinical workflow. The rationale for introducing the concept of an enhanced effective thermal conductivity *k*_eff_ is that the most important effect of fluid flow on the temperature distribution is more efficient heat transport. The main advantage of this simplification is that the value of the thermal conductivity can be adjusted without any further change to existing hyperthermia treatment planning software. Similar ideas, directed mostly to the heat transport in blood vessels, have been reported in the literature [59,60]. The different values of *k*_eff_/*k* provide the possibility to study the effect of this increased effective thermal conductivity *k*_eff_, and to determine whether an appropriate value could approximate the advanced fluid dynamics model.

### 2.3. Analysis

The treatment plans, i.e., the set of phase and amplitude settings for each antenna, were obtained by SAR-optimisation, but evaluated by the temperature distribution; consequently, the perceived quality of a single SAR-based set of device settings, depends on the choice of the model used to compute the temperature distribution. The treatment plans were analysed by determining the *T*_90_, *T*_50_ and *T*_10_ at steady state for the target volume, that is, the temperature exceeded by 90%, 50% and 10% of the target volume, respectively. The target volume was split into the tumour and a 1 cm margin around the tumour, which may cover regions of special interest, such as micrometastases or non-resectable tumour residue. The tumour margin is analysed separately for solid tissues and CSF. For the grey and white matter outside the tumour margin, we determined the *V*_41_, *V*_42_, and *V*_43_, defined as the volume in which the temperature exceeds 41 °C, 42 °C and 43 °C, respectively. The same parameters were determined for the CSF outside the tumour margin. 

Hot spots were defined as regions with *T* > 44.0 °C within the macroscopic tumour including tumour cysts, and as regions with *T* > 43 °C outside the macroscopic tumour [61,62]. The hot spots were analysed by determining their cumulative volume for the CSF and the solid tissues separately and together. We used the Dice coefficient [63] to quantify the amount of overlap between hot spots in the fluid model on the one hand and in the solid and high-*k* models, respectively, on the other hand. The Dice coefficient of two sets *A* and *B* is defined as 2 *A*∩*B* / (|*A*| + |*B*|), i.e., the intersection of the two sets divided by the average size of the sets, which ranges from 0 for disjoint sets to 1 for identical sets. 

In the second part of the analysis, we evaluated the difference between the simulation methods, and determined the median error, interquartile range and 95%-interval of the error of the solid and high-*k* methods relative to the fluid method. This part of the analysis shows how well each solid model approximates the fluid model.

## 3. Results

The pre-operative mesh comprised 2.0 × 10^6^ cells in the CSF region (340 mL) and 3.9 × 10^6^ cells for the solid regions; for the post-operative case, these numbers were 2.8 × 10^6^ fluid cells (448 mL CSF) and 3.7 × 10^6^ cells for the solid regions. Typical computation times per second simulated time were between 25 s and 97 s for the solid and high-*k* cases, and about 975 s and 1788 s for the pre-operative and post-operative fluid cases, respectively, using a single core of a normal desktop personal computer (Intel Xeon E5-1603 at 2.80 GHz processor, 16 GB RAM). Computation times may have been affected by other processes running on the same computer, but are indicative of the very large difference in computation time between the fluid model and the solid models. 

**Figure 2:**
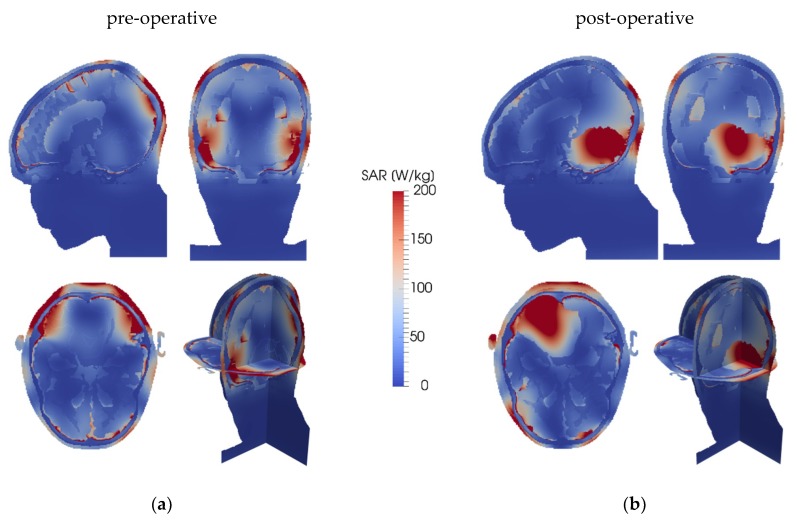
SAR distribution in the head model. The skull is clearly recognisable by its low energy uptake. Underneath the skull is typically a thin layer of CSF, which tends to have a high energy uptake. High SAR regions close to the head surface correspond with antennas with a high power output. The panels show planes through the tumour’s centre of gravity in the left-right (top-left), anterior-posterior (top-right), and cranial-caudal (bottom-left) directions, and a 3D view (bottom-right), for (**a**) the pre-operative case; (**b**) the post-operative case. The tumour is (faintly) outlined.

**Figure 3:**
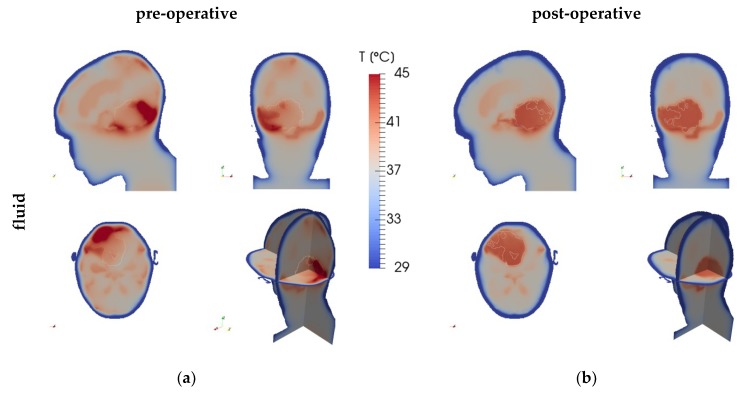

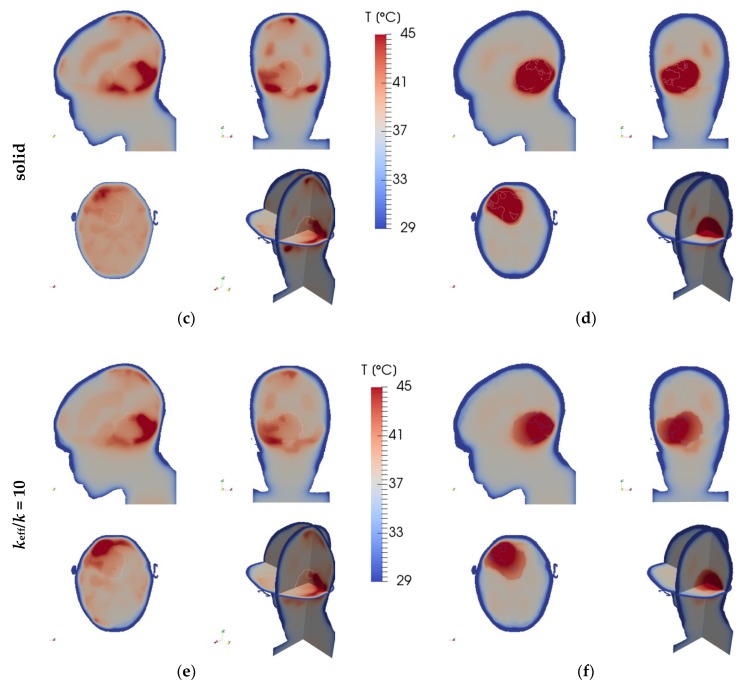
Temperature distributions for (**a,c,e**) the pre-operative and (**b**,**d,f**) post-operative cases, as predicted by (**a,b**) the fluid model, (**c,d**) the solid model, and (**e,f**) the high-*k* (*k*_eff_/*k* = 10) model, per case based on the same SAR distribution (Figure 2). The panels show planes through the tumour’s centre of gravity in the left-right (top-left), anterior-posterior (top-right), and cranial-caudal (bottom-left) directions, and a 3D view (bottom-right). The tumour (region) is outlined.

**Figure 4:**
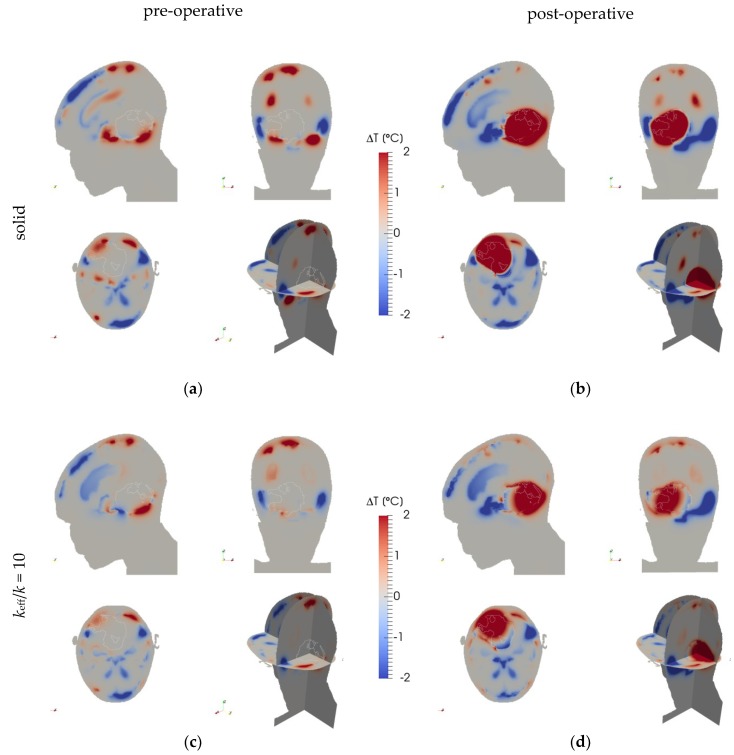
Temperature difference of (**a,b**) the solid and (**c,d**) the high-*k* (*k*_eff_/*k* = 10) model with the fluid model for (**a,c**) the pre-operative and (**b,d**) post-operative cases. The panels show planes through the tumour’s centre of gravity in the left-right (top-left), anterior-posterior (top-right), and cranial-caudal (bottom-left) directions, and a 3D view (bottom-right). The tumour (region) is outlined.

Figure 2 depicts the SAR distribution in the pre-operative and post-operative cases, while Figure 3 shows the resulting temperature distributions for the fluid, solid, and high-*k* models, and Figure 4 shows the temperature differences between the fluid model and the solid and high-*k* models, respectively. The high-*k* figures are for *k*_eff_/*k* = 10.

**Figure 5:**
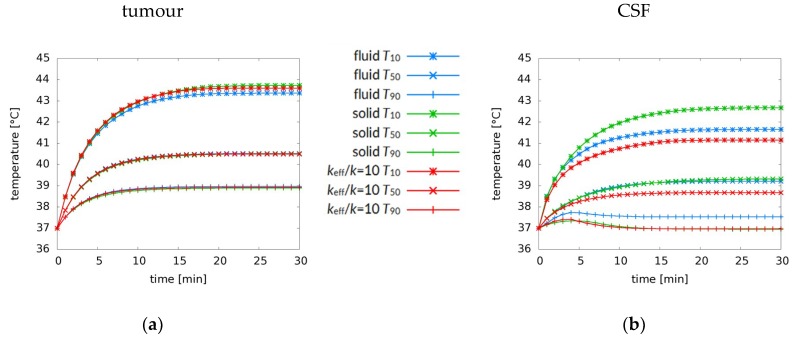
Development of *T*_90_, *T*_50_, and *T*_10_ over time for (**a**) the tumour and (**b**) the CSF (pre-operative case) for the fluid model (blue), the solid model (green) and the high-*k* model (*k*_eff_/*k* = 10) (red). Initially, the graphs are virtually similar, but after a few minutes the fluid behaviour results in a different temperature profile. While the temperature increase starts slowing down after about five minutes, it takes about 25 min to reach the equilibrium situation. Figure (b) also shows the cooling effect of the water bolus on the *T*_90_.

We simulated the first 30 min (1800 s) of a treatment. While a typical hyperthermia treatment may last between 60 and 90 min, an equilibrium is reached long before then. The development over time of the *T*_90_, *T*_50_, and *T*_10_, shown for the pre-operative case in Figure 5 for (a) the tumour and (b) the CSF, confirms that an equilibrium is reached within these 1800 s. The cooling effect of the water bolus needs some time to penetrate into the body, which causes the delayed decrease of the *T*_90_ in the CSF after an initial increase. The absence of such a delayed decrease in the tumour graph indicates that tumour temperature is not affected by the water bolus temperature. An analysis of the complete temperature distribution confirms that temperatures below 37 °C are only found in the outer 0.5–1.0 cm of the skull.

**Figure 6:**
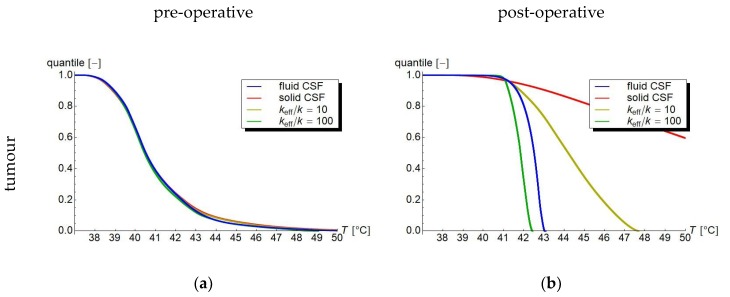

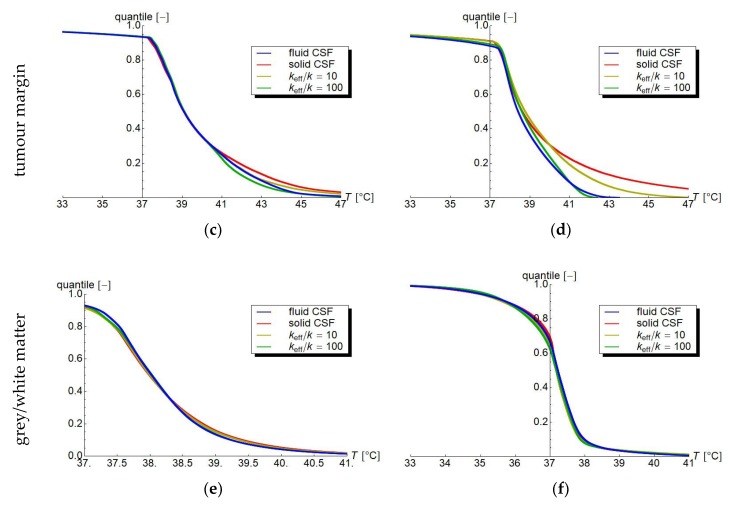
Temperature volume histograms showing volume quantile versus temperature for (**a,b**) the tumour (region), (**c,d**) the tumour margin (tumour margin), and (**e,f**) the grey/and white matter. (**a,c,e**) In the pre-operative case, these temperature volume histograms are fairly similar for the fluid, solid, and high-*k* models, although the solid and high-*k* models tend to overestimate high temperature volumes. (**b**,**d,f**) Post-operatively, the situation is similar for the white and grey matter, and more pronounced for the tumour margin. For the CSF-filled volume where the tumour has been excised, the differences are much larger. The fluid model reflects the efficient convective mixing leading to a relatively homogeneous temperature in the fluid volume, the solid model has no way to efficiently dispose of the generated heat and, therefore, greatly overestimates the temperature. The high-*k* models vary between those two, and may overestimate heat removal for large values of *k*_eff_/*k*, underestimating temperatures.

**Figure 7:**
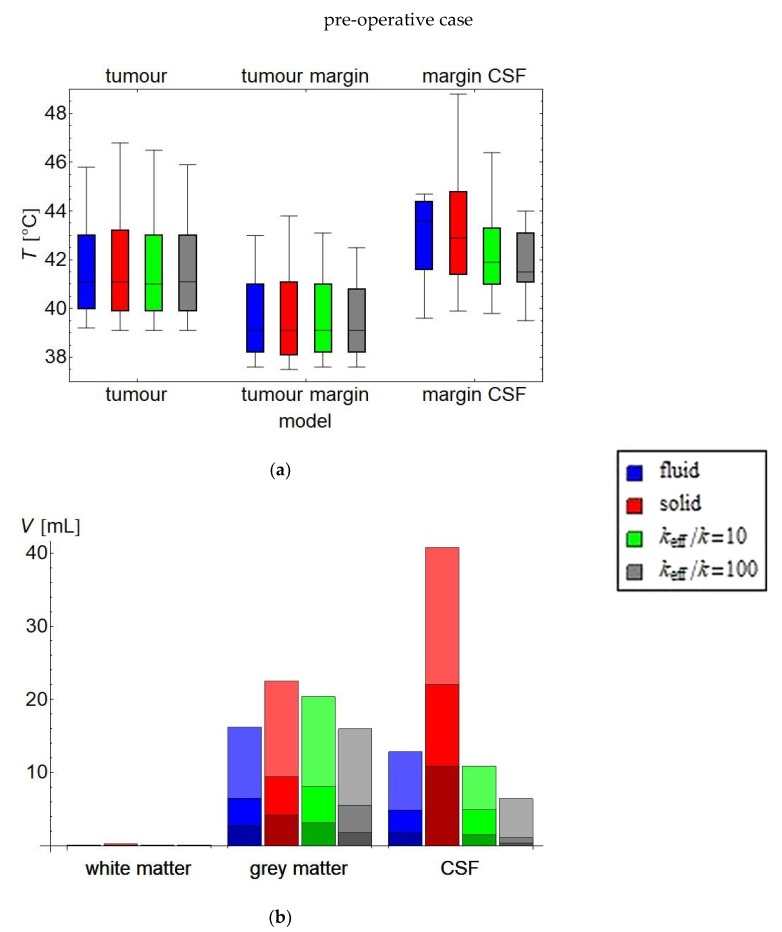
Analysis of treatment plan parameters for the post-operative case. (**a**) shows the temperature distribution in the treatment target: box: inter-quartile range with median temperature *T*_50_ indicated; whiskers: *T*_90_ and *T*_10_, i.e., the temperature exceeded by, respectively, 90% and 10% of the volume. For the treatment target, a temperature in the range 42–43 °C is clinically considered optimal. (**b**) shows for the healthy tissue the volume exceeding, respectively, 41, 42 and 43 °C (a darker shade representing a higher temperature). Clinically, these volumes should be as low as possible. Total volume: white matter = 125 mL; grey matter = 1007 mL; CSF = 340 mL; tumour = 92 mL. The exact numbers are provided in Table S1.

**Figure 8:**
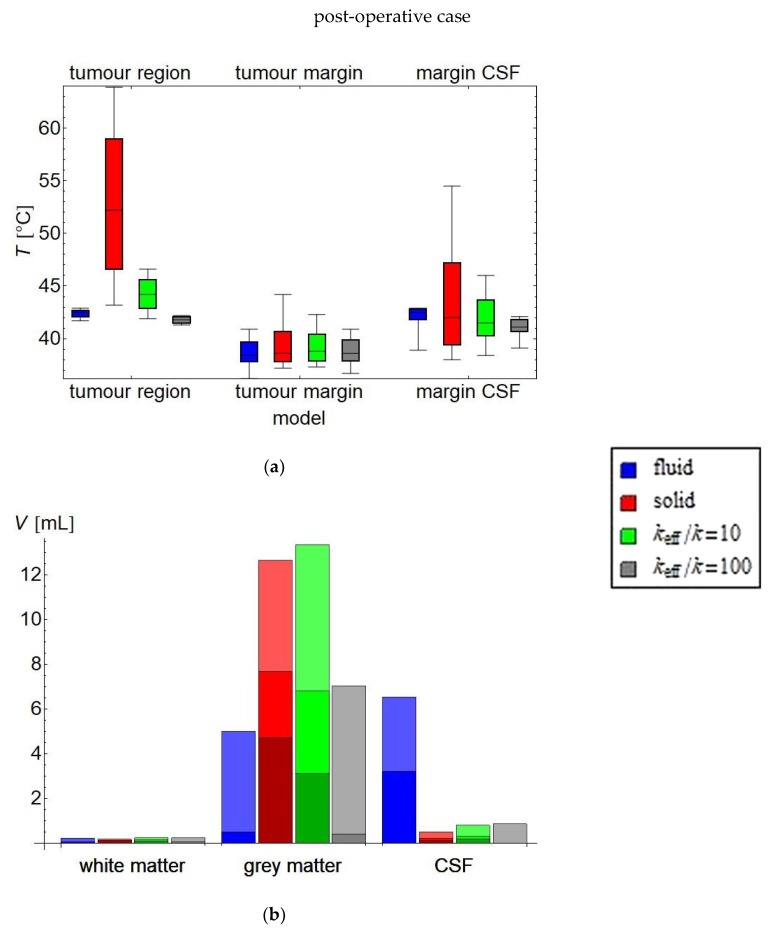
Analysis of treatment plan parameters for the pre-operative case. (**a**) shows the temperature distribution in the treatment target: box: inter-quartile range with median temperature *T*_50_ indicated; whiskers: *T*_90_ and *T*_10_, i.e., the temperature exceeded by, respectively, 90% and 10% of the volume. For the treatment target, a temperature in the range 42–43 °C is clinically considered optimal. (**b**) shows for the healthy tissue the volume exceeding, respectively, 41, 42, and 43 °C (a darker shade representing a higher temperature). Clinically, these volumes should be as low as possible. Total volume: white matter = 125 mL; grey matter = 1007 mL; CSF = 448 mL. The exact numbers are provided in Table S2.

**Figure 9:**
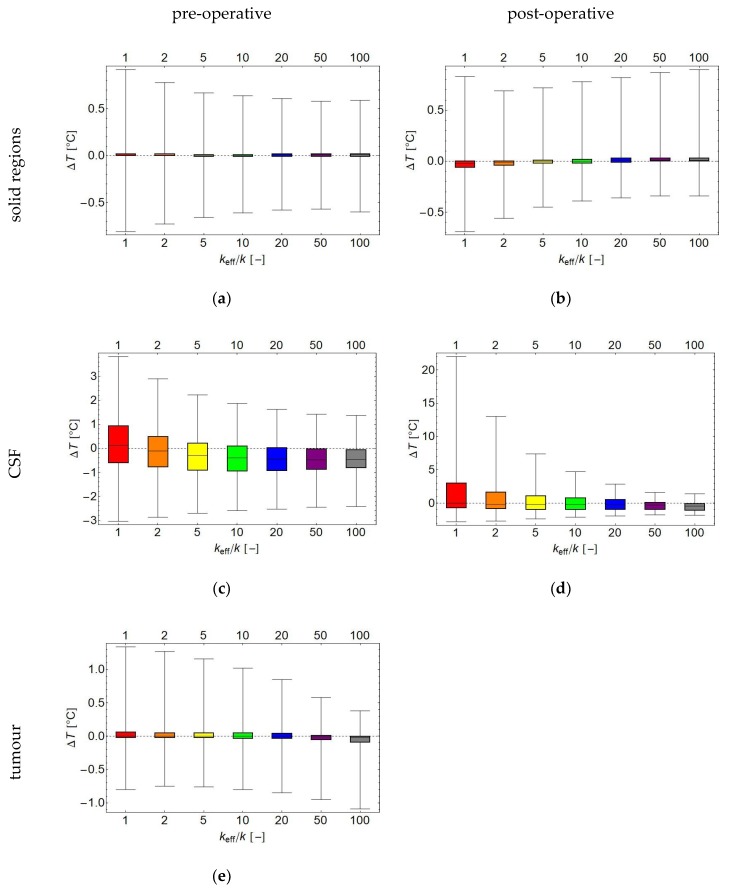
Box whisker plots of the temperature differences for the solid regions except tumour (**a**,**b**), the CSF (**c,d**) and the tumour (**e**) as predicted by the fluid model and the solid model (*k*_eff_/*k* = 1*)* and the high-*k* models for the pre-operative, respectively the post-operative case. A high-*k* model with higher *k*_eff_ tends to slightly underestimate the temperature, but has smaller error bars. Horizontal axis: *k*_eff_/*k*, vertical axis temperature in °C.

Figure 6, Figure 7, Figure 8 and Figure 9 provide temperature results for the tumour including tumour cysts, respectively the tumour region, the tumour margin, and the CSF. The tumour margin is defined as a 1 cm margin around the (former) tumour and is analysed separately as it is often a clinically relevant region: in the case of, e.g., glioblastoma, there are usually infiltrations or micrometastases in this region, while in the case of medulloblastoma, where micrometastases are not limited to a margin around the macroscopic tumour but may spread through the entire central nervous system, there are often non-resectable tumour residues. Unless otherwise specified, the high-*k* models are represented by the *k*_eff_/*k* = 10 model.

Figure 6a–f show the temperature volume histograms, i.e., graphs depicting the quantile of the volume heated to at least a given temperature, for the tumour, respectively or tumour region, the tumour margin, and grey and white matter. For the pre-operative case, the graphs of the four models are very similar: the main difference is that the fluid model shows fewer severe hot spots in the tumour margin than the solid and *k*_eff_/*k* = 10 models. Additionally, the *k*_eff_/*k* = 100 model misses some relatively mild hot spots. In the post-operative case, these differences are much more pronounced, especially for the tumour region. Here, the fluid model shows a much more homogeneous profile compared to the solid and *k*_eff_/*k* = 10 models, due to the effective convective mixing in the fluid volume. The high-*k* models are closer to the fluid model than the solid model, but still underestimate the mixing effect, and, for larger *k*_eff_, introduce a systematic cooling error.

The main thermal parameters for evaluation of the treatment plan are summarised in Figure 7 and Table S1 for the pre-operative case and in Figure 8 and Table S2 for the post-operative case. For the pre-operative case, the *T*_90_ and *T*_50_ in the tumour and in the solid tissues in the tumour margin may be considered equivalent, as they differ at most 0.1 °C As *T*_90_ and *T*_50_ are most indicative of clinical effect, all three simulation methods predict a comparable efficacy of this treatment plan; i.e., of the given set of phase and amplitude settings for the antennas. All three methods predict a hot spot in the tumour, although they differ in the severity (45.8 °C for the fluid model, 46.4 °C for the high-*k* model, and 46.8 °C for the solid model). The most pronounced differences are for the *T*_10_ in the CSF surrounding the tumour: a moderate 44.7 °C hot spot for the fluid model, versus a 46.3 °C hot spot for the high-*k* model and an even higher 48.8 °C for the solid model. In the grey and white matter analysis, the results are qualitatively the same for all models, with the exception of the CSF outside the tumour margin, where the solid model predicts 10.8 mL of CSF ≥ 43 °C, compared to only 1.8 mL for the fluid model and 1.2 mL for the high-*k* model. 

In the pre-operative case, the solid model predicts considerably more and larger hot spots in the solid tissue outside the tumour than the fluid model (Dice coefficient 0.68); while even the best performing high-*k* model (*k*_eff_/*k* = 20) misses the smaller hot spots predicted by the fluid model, and overestimates the size of the hot spot between the tumour and the back of the skull (Dice coefficient 0.77). As *k*_eff_ increases, the amount of incorrectly predicted hot spots (false positives) reduces, while the missed hot spots (false negatives) increase in volume. The hot spots in the CSF are again overestimated by the solid model, but increasingly missed with increasing *k*_eff_, so that the *k*_eff_/*k* = 2 model actually performs best with regards to hot spots in the CSF (Dice coefficient 0.67). A full overview of the hot spot volumes and Dice coefficients is given in Table 2.

In the post-operative case, the differences in the treatment plan analysis are larger than in the pre-operative case. The most notable difference is found in the *T*_10_ (i.e., highest temperatures) for the target regions and the *T*_50_ for the circumtumoral CSF. In particular, the solid model predicts a *T*_50_ of 52.2 °C for the circumtumoral CSF, compared to an almost acceptable 44.2 °C for the *k*_eff_/*k* = 10 model and a safe and desirable 42.5 °C for the fluid model. The *T*_90_ for the solid tumour margin is predicted as 40.9 °C by the fluid model, 42.3 °C by the *k*_eff_/*k* = 10 model, and 44.2 °C by the solid model. 

The fluid model predicts almost no hot spots in the post-operative case, while the solid model predicts a large hot spot in the former tumour region, and small hot spots immediately around it. As *k*_eff_ increases, the hot spots inside and surrounding the CSF decrease, until they disappear for *k*_eff_/*k* ≥ 50. As the reference hot spot volume is practically zero, the Dice coefficients are all < 0.1 for the post-operative case and not included in Table 2.

Figure 9a–e summarise the difference of the solid and high-*k* models with the fluid model for the pre-operative and post-operative cases, respectively, in a set of box whisker plots showing the median, interquartile range, and 95%-interval. For the pre-operative case, the median temperature difference and interquartile range are all well within ±0.1 °C. However, the solid model temperature error for the healthy tissues has a larger spread than the high-*k* models: the 95% interval decreases from (−0.8 °C, +0.9 °C) for the solid model to ±0.6 °C for *k*_eff_/*k* = 50. This implies that the high-*k* models give a better approximation to the fluid dynamics (with, in this case, an optimum for *k*_eff_/*k* = 50), but may still be insufficiently precise for many applications. For the grey and white matter, a similar pattern emerges, but with generally smaller inaccuracies. For the temperature of the CSF, both outside the target region, and in particular inside the target region, the predictions by the solid and model are even less reliable; for example, the 2.5th and 97.5th percentile of the temperature difference for the CSF in the target region are −3.85 °C and 9.05 °C, respectively; these values move towards zero for increasing *k*_eff_, and are −3.34 °C and 1.37 °C for the *k*_eff_/*k* = 100 model. The CSF temperature, both inside and outside the target region, is generally underestimated for higher *k*_eff_, with the interquartile range between roughly −1 °C and 0 °C.

For the post-operative case, the results are essentially similar, but magnified. The largest temperature deviations are, unsurprisingly, obtained for the solid model in the CSF-filled (former) tumour region, where the mean ± SD temperature difference is 10.6 ± 7.7 °C and the median temperature difference is 9.8 °C. For the *k*_eff_/*k* = 10 model, these numbers have shrunk to 1.8 ± 1.9 °C and 1.8 °C, respectively. For the grey and white matter, the difference between the solid and high-*k* models on the one hand and the fluid model on the other, are much smaller. The mean and median differences are all smaller than 0.1 °C, and the standard deviation varies from 0.2 °C (white matter, *k*_eff_/*k* = 10 model) to 0.6 °C (grey matter, solid model).

In general, a higher *k*_eff_ leads to a lower estimated temperature in the CSF, which may easily lead to an underestimation of the fluid temperature. This is seen in both the pre-operative (Figure 9c) and post-operative (Figure 9d) cases, where the median temperature difference decreased from +0.1 °C for the solid model to −0.5 °C for *k*_eff_/*k* ≥ 20. At the same time, the range of the errors decreases, and seems to reach a plateau for *k*_eff_/*k* ≥ 50.

**Figure 10:**
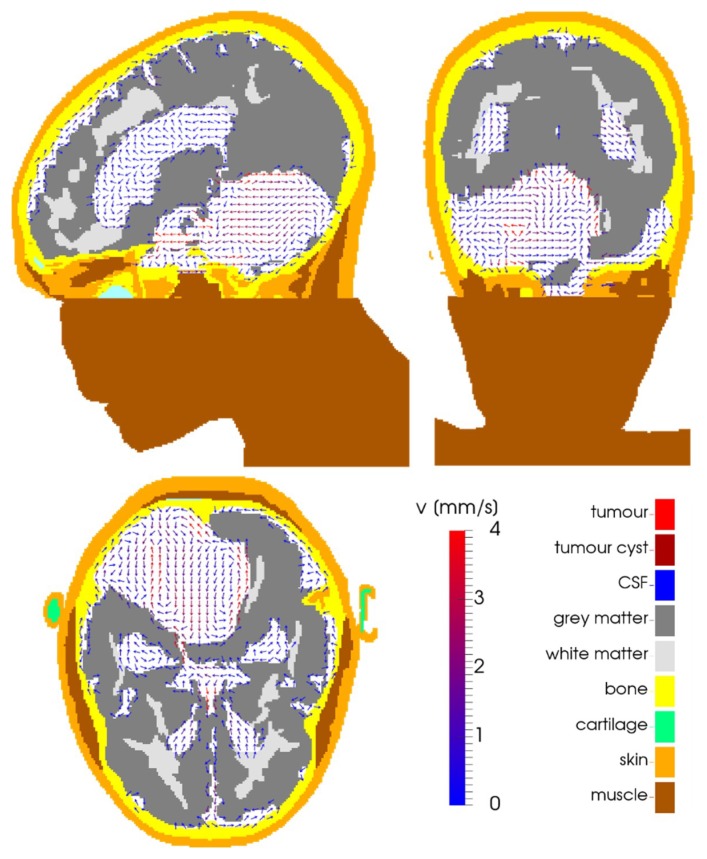
For the post-operative case, the arrows show the direction of the CSF flow in the depicted planes, while the colour of the arrows indicates the magnitude of the flow velocity, as indicated by the colour bar. The solid tissues are colour-coded according to tissue type, using the same colour map as Figure 1b. The panels show planes through the tumour’s centre of gravity in the left-right (top-left), anterior-posterior (top-right), and cranial-caudal (bottom-left) directions.

Figure 10 shows the direction and velocity of the flow in the CSF in the post-operative case.

## 4. Discussion

This study evaluated the importance of fluid dynamics modelling for treatment planning of local/loco-regional microwave hyperthermia treatments of brain tumours. As long as there are no large contiguous fluid volumes, CSF flow has a mostly minor influence on the temperature distribution in the human brain during microwave hyperthermia treatment. The differences are most noticeable in the CSF itself, and almost negligible in the tumour. For the pre-operative case, the differences in plan evaluation parameters between the three models are small (Figure 7 or Table S2), although locally the predicted temperature differences may be several degrees (Figure 9). These large differences appear mostly in regions where the temperatures are not very high, such as the CSF layer directly beneath the skull, which is not relevant as a treatment target and where, due to the vigorous surface cooling, there is no substantial danger for hot spots. The cooling effect of the water boli at 10 °C penetrates about 1 cm into the skull, which may be sufficient in some places to reach a CSF region. In such places, the CSF may spread the cooling effect over a larger volume. The models with increased effective conductivity substantially consistently perform better than the solid model with unadjusted thermal conductivity in the CSF. 

For the post-operative case, which for medulloblastoma will be the most likely clinical application, the evaluation parameters of the treatment plan (given in Figure 8 or Table S2) evaluated using the fluid model, appear to imply a very satisfactory treatment. The solid model becomes unreliable in and around the CSF regions, vastly overestimating the temperature in the CSF regions, especially the large fluid volume where the tumour has been excised. This is due to the lack of efficient heat transport by perfusion and convection, which combine to result in a large heat accumulation. In this region, the high-*k* models are substantially better approximations than the unadjusted solid model. The *T*_10_ still tends to overestimate the CSF temperature, in particular in the target region, and only by a modest amount; in general, overestimation of the *T*_10_ would be preferred over underestimation, as it is related to the risk of healthy tissue damage through hot spots. It must be noted, however, that the model used for post-operative is not completely realistic. For example, blood may mix with the CSF, altering the dielectric and physical properties of the fluid. Additionally, the region around the excised volume may deform, affecting flow patterns and the temperature distribution. Therefore, for clinical practice, it is advisable to obtain a post-operative scan for post-operative hyperthermia treatment planning, though the models in our study provide important basic insights in the difference between the pre-operative and post-operative cases.

Increasing the value for *k*_eff_ increases the precision of the temperature simulation, in particular for the CSF itself; naturally, this effect is more pronounced in the post-operative case. However, as the median temperature is reduced, the median error changes from +0.1 °C to −0.4 °C. Increasing the value for *k*_eff_ has a limited effect for *k*_eff_/*k* ≳ 20. This is in line with the results reported by Yuan et al. [58] for the urinary bladder, who found that when using *k*_eff_ > 10 W/(m K) for the bladder, the temperature difference between simulated and measured bladder temperatures tended to reach a plateau. A solid foundation for a fixed choice of *k*_eff_ for the CSF requires a larger sample of cases simulated with the fluid model. Another solution might involve adding a (small) heat sink term to the CSF model analogous to the blood perfusion heat sink term in Pennes’ equation, which would serve to reduce the extremely high temperatures erroneously predicted by the solid model(s). Results of bladder studies show that such a term might improve the accuracy of the results [48]. Another alternative might involve a temperature dependent or non-isotropic value for *k*, with a high value for *k* in the direction perpendicular to the direction of gravity, and a higher value for *k* in de upward direction than in the downward direction; such a non-isotropic *k* would be quite straightforward to implement in a finite difference model. As the amount of heat transport by natural convection depends on the actual temperature differences in the fluid, a more sophisticated *k*_eff_ model might implement a *k*_eff_ that is proportional to temperature differences within the fluid volume, e.g., by relating *k*_eff_ to the local temperature gradient.

Uncertainties in the temperature dependency of the used parameters may have caused errors in the prediction. There is little information in the literature about the relationship between temperature and various physical parameters; this is most relevant for perfusion, but also for, e.g., electric conductivity and permittivity. In this study, an increased perfusion value was used for muscle (which is hardly present in and around the treatment area), but not for other tissue types, most notably not for grey and white matter. Based on experiments with monkeys and dogs in the late eighties and early nineties, cerebral perfusion is estimated to increase about 2.5-fold in healthy tissue and about 1.5-fold in tumour tissue when the brain temperature is increased to hyperthermic temperatures [64,65,66]. Implementation of such dynamic temperature dependent perfusion values would lead to relatively selective heating of the tumour, and less risk of potential hot spots. This is also true for small CSF volumes which release more of their heat to better perfused tissues, but the problem of avoiding CSF overheating remains for larger volumes.

The Chalmers Hyperthermia Helmet is the only brain hyperthermia applicator designed specifically for paediatric use. The fluid model simulations in this study show that the Chalmers Hyperthermia Helmet is capable of obtaining an adequate tumour temperature, without inducing unacceptable hot spots. The relatively large number of antennas (up to 16) and the wide frequency range (300–900 MHz) of the antennas, allow for a high degree of spatial steering [38,54]. Hot spots may threaten to appear at tissue interfaces with inhomogeneities in dielectric and thermal properties; in practice, these occur mostly on CSF boundaries. The CSF may then serve to efficiently spread the excess heat over a larger volume, reducing the risk of hot spots.

The results from this paper regarding the influence of CSF on the temperature distribution, apply equally to various alternative hyperthermia applicators that are being or have been developed for heating brain tumours, albeit that they are designed for adults, and not for paediatric applications. A group in Berlin is developing a high field-strength MR-compatible applicator comprising a ring of 16 bowtie antennas [67]. Their initial SAR simulation results look promising for relatively small tumours, but they have not performed temperature simulations. However, temperature-based planning, or at least temperature-based plan verification is preferable, in our view, as there is no direct correlation between SAR and temperature between different tissue types. A 72-channel 915-MHz system (HYPER-crown) is currently being developed specifically for brain tumours by a commercial party [68], similar to the existing HYPER-collar [69]. Treatment planning for these devices is mostly SAR-based [69,70], but treatment monitoring and treatment evaluation may use temperature simulations. Temperature simulation data for brain tumours are not available, but temperature simulations for head and neck sarcomas for treated with the HYPER-collar showed an error of about 2 °C relative to measurements during treatment using patient-group optimised parameters [71,72].

The discussed brain hyperthermia applicators (including the Chalmers Hyperthermia Helmet) are all MR-compatible, so that, in principle, temperature may be monitored during treatment using MR thermometry. However, especially in a post-operative scenario with a large fluid-filled pocket, these measurements may be unreliable in and around the CSF-filled pocket due to motion artefacts; this is similar to MR-thermometry in and around the urinary bladder [73], for which an MR thermometry accuracy of 1.86 ± 1.20 °C has been reported [74]. Therefore, additional forms of treatment guidance would still be needed, such as numerical temperature simulation; if possible, supplemented with, preferably invasive, thermometry. If MR-guidance is used, SAR and temperature simulations may be further improved by obtaining patient-specific (and possibly temperature dependent) dielectric parameters [75].

Fluid dynamics modelling is also of crucial importance for other brain heating techniques, such as magnetic nanoparticles. The first clinical results of magnetic nanoparticle heating for glioblastoma have been reported, which showed that controlled heating is quite challenging [20]. Although aiming for hyperthermic temperatures of 43 °C, ablative temperatures of up to 82 °C have been recorded during treatment. If these nanoparticles are transported by the CSF, this adds an additional importance to model fluid behaviour in order to track and/or predict the distribution of these nanoparticles.

In the end, the exact requirements on the heating pattern and the accuracy with which the treatment can be predicted or reconstructed, depend primarily on the relevant hyperthermia mechanism, which in turn may depend on the primary treatment modality: radiotherapy and/or chemotherapy. For example, for certain chemotherapy applications, it may be particularly important to have accurate knowledge whether sufficiently high temperatures have been reached to open the blood brain barrier. When drugs are delivered using thermosensitive liposomes (nanocarriers that release their payload above a certain activation temperature), it is especially important to know the volume where the activation temperature of the liposomes has been obtained. In the case of radiotherapy, it may both be relevant to know whether the temperature was adequate to inhibit fast DNA damage repair, and to know the complete thermal dose for slow DNA damage repair. Regardless of treatment modality, accurate knowledge of presence or absence of hot spots in healthy tissue is always important.

Using the current set-up, simulating the fluid simulations took more than 15 min (pre-operative) or almost 30 min (post-operative) of real time for each second of simulation time. Evidently, this makes the fluid dynamics model in its current form unpractical for clinical use. A lower resolution would greatly speed up the computations, but this may negatively affect the accuracy in both the spatial and temperature domains. In a well-controlled phantom experiment, we showed that the fluid solver remains accurate when using 2.5 mm-based cells instead of 1 mm-based cells [47], but this may result in losing smaller CSF canals. A possible solution might be to use a high resolution around the tumour location and in regions with a high SAR. As for clinical applications steady state temperatures are of central importance, a steady state solver (e.g., based on the SIMPLE algorithm [57]) might be more efficient. Temperature-based optimisation will, most likely, remain unfeasible, but the increase in speed may be sufficient for treatment plan validation. Given the current practical limitations on computation times and the relatively good approximations of the high-*k* model, an acceptable workflow might comprise doing the amplitude and phase optimisation using a computationally efficient high-*k* model (with *k*_eff_/*k* equal to about 10–20), possibly with relaxed constraints on the temperature in the CSF. Preferably, such a plan should be validated with a final check using a (steady-state) fluid dynamics model.

## 5. Conclusions

Heating the brain is a promising (adjuvant) treatment option, but also challenging. For estimating thermal dose parameters for the tumour, physically correct fluid modelling may not be required. For healthy tissue, however, not taking fluid behaviour into account may lead to erroneous predictions of severe hot spots. Unfortunately, the applied thermophysical fluid model is, in its current state, not fast enough for routine clinical use. As an intermediate solution, a temperature that is accurate to within about 1 °C may be obtained for tissues (excluding CSF itself) by modelling CSF as a special solid with an increased effective thermal conductivity; this may be sufficiently accurate for clinical use, provided stringent safety margins are maintained. The optimal value for the effective thermal conductivity depends on the individual case, but a roughly 10–20-fold increase appears to best balance the different effects of convective heat transport on the temperature distribution.

## Figures and Tables

**Table 1:** Dielectric and thermal parameter values at 450 MHz for the various tissue types used in the simulations: electrical conductivity (σ [S/m]), relative permittivity (ε_r_ [-]), density (ρ [kg/m^3^]), specific heat capacity (*C_t_* [J/(kg K)]), thermal conductivity (*k* [W/(m K)]) and perfusion coefficient (ω *C_b_* [W/(m^3^ K)]), being the product of the volumetric perfusion rate (ω [kg/(m^3^ s)]) and the specific heat capacity of blood (*C_b_* = 3600 W/(m^3^ K). The last column indicates whether a tissue was modelled using convective flow simulations (“yes”), or using Pennes’ equation (“no”). Parameters were obtained from the IT’IS database [50], Gabriel and Gabriel [51], and Wang et al. [52]. The perfusion value of muscle has been increased four-fold to account for vasodilation at hyperthermic temperatures, in accordance with Kok et al. [53].

Tissue Type	σ (S/m)	ε_r_ (–)	ρ (kg/m^3^)	*C_t_* (J/(kg K))	*k* (W/(m K))	ω *C_b_* (W/(m^3^ K))	Flow
tumour	0.84	56.64	1056	3700	0.57	22,545	no
tumour cyst	0.84	56.64	1056	3700	0.57	0	no
cerebrospinal fluid	2.28	70.95	1007	4200	0.62	0	yes
grey matter	0.76	56.48	1038	3700	0.57	45,090	no
white matter	0.46	41.43	1038	3600	0.50	15,925	no
muscle	0.93	75	1050	3639	0.56	12,960	no
bone	0.095	13.03	1990	1300	0.40	3400	no
cartilage	0.60	44.94	1097	3500	0.47	9000	no
skin	0.71	45.70	1125	3500	0.42	8065	no
vitreous humour	1.54	69.00	1009	4200	0.60	0	no
air	0	1	1.293	10,000	0.024	0	no
bolus water	0.070	83.83	1000	4180	0.60	0	no

**Table 2:** Hot spot volume and overlap as predicted by the various models and cases are shown. For the pre-operative case, the volume of the hot spots (in mL) in healthy tissue is given, the overlap with the volume according to the convective fluid model, and the Dice coefficient of the overlap; this is done separately for the solid tissues (except tumour and tumour cyst), the CSF, and the combination. The model having the highest Dice coefficient is rendered in boldface. For the post-operative case, only the volumes (in mL) are given, as the convective fluid model predicts hardly any hot spots and all Dice coefficients are <0.1.

Case	Region	Parameter	Fluid	Solid	2 *k*	5 *k*	10 *k*	20 *k*	50 *k*	100 *k*
pre-op	solid	volume	22.31	36.46	30.45	24.84	22.29	**20.55**	17.43	14.26
		overlap	22.31	20.00	19.29	17.71	16.88	**16.42**	15.11	13.14
		Dice	1.00	0.68	0.73	0.75	0.76	**0.77**	0.76	0.72
	CSF	volume	23.94	29.37	**21.39**	16.12	13.20	12.61	12.57	11.68
		overlap	23.94	17.08	**15.15**	12.69	11.30	11.56	11.62	10.78
		Dice	1.00	0.64	**0.67**	0.63	0.61	0.63	0.64	0.61
	total	volume	46.25	65.83	51.85	40.96	35.49	**33.17**	29.99	25.94
		overlap	46.25	37.08	34.45	30.40	28.18	**27.99**	26.73	23.91
		Dice	1.00	0.66	0.70	0.70	0.69	**0.70**	0.70	0.66
post-op	solid	volume	0.23	19.76	17.37	14.33	10.59	5.60	0.64	0
	CSF	volume	2.74	125.11	120.91	112.89	103.80	84.28	24.95	0
	total	volume	2.97	144.87	138.27	127.22	114.38	89.88	25.59	0

## Supplementary Materials

The following are available online at https://www.mdpi.com/2072-6694/11/8/1183/s1, **Table S1.** Thermal parameters for the analysis of a single treatment plan (ergo the same SAR-distribution), according to three different models; results for the pre-operative case. Treatment target evaluation parameters *T*_90_, *T*_50_ and *T*_10_ are given, i.e., the temperature exceeded by, respectively, 90, 50 and 10% of the volume (temperatures in the range 42–43 °C are generally considered clinically optimal temperatures). For the healthy tissue, the volume exceeding, respectively, 41, 42 and 43 °C is given (lower volumes are clinically preferred). Total volume: white matter = 124.669 mL; grey matter = 1006.547 mL; CSF = 339.982 mL; tumour = 92.031 mL. **Table S2.** Thermal parameters for the analysis of a single treatment plan (ergo the same SAR-distribution), according to three different models; results for the post-operative case. Treatment target evaluation parameters *T*_90_, *T*_50_ and *T*_10_ are given, i.e., the temperature exceeded by, respectively, 90, 50 and 10% of the volume (temperatures in the range 42–43 °C are generally considered clinically optimal temperatures). For the healthy tissue, the volume exceeding, respectively, 41, 42 and 43 °C is given (lower volumes are clinically preferred). Total volume: white matter = 124.669 mL; grey matter = 1006.547 mL; CSF = 447.709 mL.
