# Supplementary material for: Hyperthermia Treatment Planning Including Convective Flow in Cerebrospinal Fluid for Brain Tumour Hyperthermia Treatment Using a Novel Dedicated Paediatric Brain Applicator
Source: Cancers (Basel). 2019 Aug 15;11(8):1183. doi: 10.3390/cancers11081183 (PMC6721488; doi:10.3390/cancers11081183)
Supplement: Supplementary file 1 [file cancers-11-01183-s001.pdf]

cancers

Article

Hyperthermia treatment planning including convective flow in cerebrospinal fluid for brain tumour hyperthermia treatment using a novel dedicated paediatric brain applicator

G. Schooneveldt [1,*], H. Dobšiček Trefná [2], M. Persson [2], T.M. de Reijke [3], K. Blomgren [4], H.P. Kok [1] and J. Crezee [1]

[1] Dept of Radiotherapy, Amsterdam UMC, University of Amsterdam, the Netherlands
[2] Dept of Electrical Engineering, Chalmers University of Technology, Gothenburg, Sweden
[3] Dept of Urology, Amsterdam UMC, University of Amsterdam, the Netherlands
[4] Dept of Women's and Children's Health, Karolinska Institute, Stockholm, Sweden
* Correspondence: g.schooneveldt@amsterdamumc.nl

Received: 29 June 2019; Accepted: 13 August 2019; Published: 15 August 2019

1. Supplemental Material

Table S1. Thermal parameters for the analysis of a single treatment plan (ergo the same SAR-distribution), according to three different models results for the pre-operative case. Treatment target evaluation parameters T_{90}, T_{50}, and T_{10} are given, *i.e.* the temperature exceeded by, respectively, 90, 50, and 10 % of the volume (temperatures in the range 42–43 °C are generally considered clinically optimal temperatures). For the healthy tissue, the volume exceeding, respectively, 41, 42, and 43 °C is given (lower volumes are clinically preferred). Total volume: white matter = 124.669 ml; grey matter = 1006.547 ml; CSF = 339.982 ml; tumour = 92.031 ml.

	pre-operative case								
**organ**	**fluid**			**solid**			**$k_{eff}/k = 10$**		
**target**	**T_{90} [°C]**	**T_{50} [°C]**	**T_{10} [°C]**	**T_{90} [°C]**	**T_{50} [°C]**	**T_{10} [°C]**	**T_{90} [°C]**	**T_{50} [°C]**	**T_{10} [°C]**
tumour[1]	39.2	41.1	45.8	39.1	41.1	46.8	39.1	41.0	46.5
tumour margin[2]	37.6	39.1	43.0	37.5	39.1	43.8	37.6	39.1	43.0
CSF[3]	39.6	43.6	44.7	39.9	42.9	48.8	39.8	41.9	46.3
**Healthy tissue**	**V_{41} [ml]**	**V_{42} [ml]**	**V_{43} [ml]**	**V_{41} [ml]**	**V_{42} [ml]**	**V_{43} [ml]**	**V_{41} [ml]**	**V_{42} [ml]**	**V_{43} [ml]**
white matter	0.066	0.041	0.030	0.240	0.041	0.038	0.070	0.042	0.041
grey matter	16.179	6.504	2.741	22.497	9.394	4.209	19.806	7.764	3.017
CSF[4]	12.841	4.840	1.816	40.757	22.060	10.847	10.504	4.722	1.220

[1]Including cysts; [2]i.e. a 1 cm solid tissue margin around the tumour; [3]CSF in the tumour margin; [4]CSF outside the target volume only.

Table S2. Thermal parameters for the analysis of a single treatment plan (ergo the same SAR-distribution), according to three different models results for the post-operative case. Treatment target evaluation parameters T_{90}, T_{50}, and T_{10} are given, *i.e.* the temperature exceeded by, respectively, 90, 50, and 10 % of the volume (temperatures in the range 42–43 °C are generally considered clinically optimal temperatures). For the healthy tissue, the volume exceeding, respectively, 41, 42, and 43 °C is given (lower volumes are clinically preferred). Total volume: white matter = 124.669 ml; grey matter = 1006.547 ml; CSF = 447.709 ml.

	**post-operative case**								
**organ**	**fluid**			**solid**			**k_{eff}/k = 10**		
**target**	**T_{90} [°C]**	**T_{50}[°C]**	**T_{10} [°C]**	**T_{90} [°C]**	**T_{50} [°C]**	**T_{10} [°C]**	**T_{90} [°C]**	**T_{50} [°C]**	**T_{10} [°C]**
tumour region (CSF)	38.9	42.5	42.9	38.0	42.0	54.5	38.4	41.5	46.0
tumour margin[1]	36.2	38.4	40.9	37.2	38.6	44.2	37.3	38.8	42.3
CSF[2]	41.7	42.5	42.9	43.2	52.2	63.9	41.9	44.2	46.6
**Healthy tissue**	**V_{41} [ml]**	**V_{42} [ml]**	**V_{43} [ml]**	**V_{41} [ml]**	**V_{42} [ml]**	**V_{43} [ml]**	**V_{41} [ml]**	**V_{42} [ml]**	**V_{43} [ml]**
white matter	0.222	0.050	0.0	0.163	0.121	0.109	0.231	0.133	0.085
grey matter	4.995	0.496	0.0	12.642	7.665	4.702	13.342	6.799	3.107
CSF[3]	6.531	3.196	0.0	0.503	0.216	0.122	0.789	0.313	0.183

[1] I.e. a 1 cm solid tissue margin around the tumour; [2]CSF in the tumour margin; [3]CSF outside the target volume only.

© 2019 by the authors. Licensee MDPI, Basel, Switzerland. This article is an open access article distributed under the terms and conditions of the Creative Commons Attribution (CC BY) license (http://creativecommons.org/licenses/by/4.0/).
